# Pharmacodynamic modeling of adverse effects of anti-cancer drug treatment

**DOI:** 10.1007/s00228-016-2030-4

**Published:** 2016-02-26

**Authors:** A. H. M. de Vries Schultink, A. A. Suleiman, J. H. M. Schellens, J. H. Beijnen, A. D. R. Huitema

**Affiliations:** Department of Pharmacy and Pharmacology, Antoni van Leeuwenhoek–The Netherlands Cancer Institute and MC Slotervaart, Louwesweg 6, 1066 EC Amsterdam, The Netherlands; Department of Pharmacology, Clinical Pharmacology Unit, University Hospital of Cologne, Gleueler Str. 24, 50931 Cologne, Germany; Department of Clinical Pharmacology, Antoni van Leeuwenhoek–The Netherlands Cancer Institute, Plesmanlaan 121, 1066 CX Amsterdam, The Netherlands; Science Faculty, Utrecht Institute for Pharmaceutical Sciences (UIPS), Division of Pharmacoepidemiology and Clinical Pharmacology, Utrecht University, P.O. Box 80082, 3508 TB Utrecht, The Netherlands

**Keywords:** Modeling, Pharmacodynamics, Adverse effects, Anti-cancer drug treatment

## Abstract

**Purpose:**

Adverse effects related to anti-cancer drug treatment influence patient’s quality of life, have an impact on the realized dosing regimen, and can hamper response to treatment. Quantitative models that relate drug exposure to the dynamics of adverse effects have been developed and proven to be very instrumental to optimize dosing schedules. The aims of this review were (i) to provide a perspective of how adverse effects of anti-cancer drugs are modeled and (ii) to report several model structures of adverse effect models that describe relationships between drug concentrations and toxicities.

**Methods:**

Various quantitative pharmacodynamic models that model adverse effects of anti-cancer drug treatment were reviewed.

**Results:**

Quantitative models describing relationships between drug exposure and myelosuppression, cardiotoxicity, and graded adverse effects like fatigue, hand-foot syndrome (HFS), rash, and diarrhea have been presented for different anti-cancer agents, including their clinical applicability.

**Conclusions:**

Mathematical modeling of adverse effects proved to be a helpful tool to improve clinical management and support decision-making (especially in establishment of the optimal dosing regimen) in drug development. The reported models can be used as templates for modeling a variety of anti-cancer-induced adverse effects to further optimize therapy.

## Introduction

Adverse effects are a major problem in the treatment with both cytotoxic drugs and newer targeted therapies, resulting in dose reductions, dose delays, and treatment cessation. Toxicity can impair quality of life, jeopardize treatment adherence, and necessitate dose reductions and dose delays, which can negatively affect response to treatment and outcome [1, 2]. The tendency in cytotoxic anti-cancer drug treatment is to dose drugs around the maximum tolerated dose (MTD), which assumes that the highest possible dose achieves the maximum effect [3]. Adverse effects are, therefore, frequently observed during treatment with cytotoxic drugs. Targeted therapies are expected to have less toxicity, mainly because of two reasons: (i) these therapies are specific to a tumor target and induce less off target toxicity and (ii) targeted therapies might have maximum target inhibition at lower concentrations than the MTD. The latter has led to the suggestion that targeted therapies should be dosed around the optimal biological dose (where target saturation is maximal) rather than the MTD. However, definition of the optimal biological dose is hampered by the lack of validated biomarkers for efficacy, lack of information on the relation between target binding and survival measures, and yet information on the highest possible dose remains of value [4]. As a consequence, targeted therapies are still often dosed around the MTD [2].

Adverse effects related to cancer therapy are typically graded by the National Cancer Institute’s Common Terminology Criteria for Adverse Events (NCI-CTC-AE). A conventional and common approach to analyze toxicity data is to calculate the proportion of patients that experienced a certain (severe) grade of toxicity [5]. Subsequently, these proportions can be statistically related to dosing groups, area under the plasma concentration-time curve (AUC) or other summary variables for exposure [6]. However, it is essential to have information on the dynamic relation between exposure and toxicity, which provides information on when the toxicity occurs, what the severity is over time, and if or when the adverse effect is reversed. For this purpose, quantitative models are becoming increasingly important. These models describe the time course of toxicities related to exposure, as will be described throughout this review.

Since modeling adverse effects is becoming increasingly important in anti-cancer drug treatment and drug development, an overview of existing modeling approaches can be helpful for future research. Therefore, the aim of this review is to give a perspective of modeling adverse effects of anti-cancer drugs and report several model structures that describe relationships between drug concentrations and toxicities, thereby focusing on the fixed effects of non-linear mixed effects models.

## Modeling adverse effects

### Myelosuppression

Myelosuppression is the leading dose-limiting toxicity in treatment with cytotoxic agents. Hematological toxicity consists of low leukocyte, thrombocyte, and platelet counts, potentially leading to life-threatening infections, anemia, and bleeding. Neutropenia, a subtype of leucopenia, is the most common and serious hematologic toxicity observed during treatment with cytotoxic anti-cancer drugs [7]. Myelosuppression can necessitate dose reductions and dose delays, potentially resulting in suboptimal drug exposure. Early approaches to describe hematological toxicity aimed at finding correlations between summary variables of exposure and summary variables of myelosuppression, not taking into account the complete time course of either drug concentration or myelosuppression. Survival fraction of blood cells or percentage change in blood count were typically used as summary variables for myelosuppression, whereas average drug concentration, AUC, or peak drug concentration were used to summarize exposure [8–10]. These models have major limitations such as poor predictive value and lack of description of the dynamics of toxicity.

#### Empirical models

The first models describing the complete time course of myelosuppression were empirical models. One model described the time course of leucopenia in patients treated with etoposide and used a lag time to account for the delay in the myelosuppressive effect and a cubic spline function, which represented the deviation of white blood cell (WBC) count from baseline [11]. An *E*_max_ model described the decline in WBC count from baseline, which was dependent on the effective concentration of etoposide. A similar empirical model was published for paclitaxel-induced leucopenia [12].

#### Semi-mechanistic models

**Fig 1:**
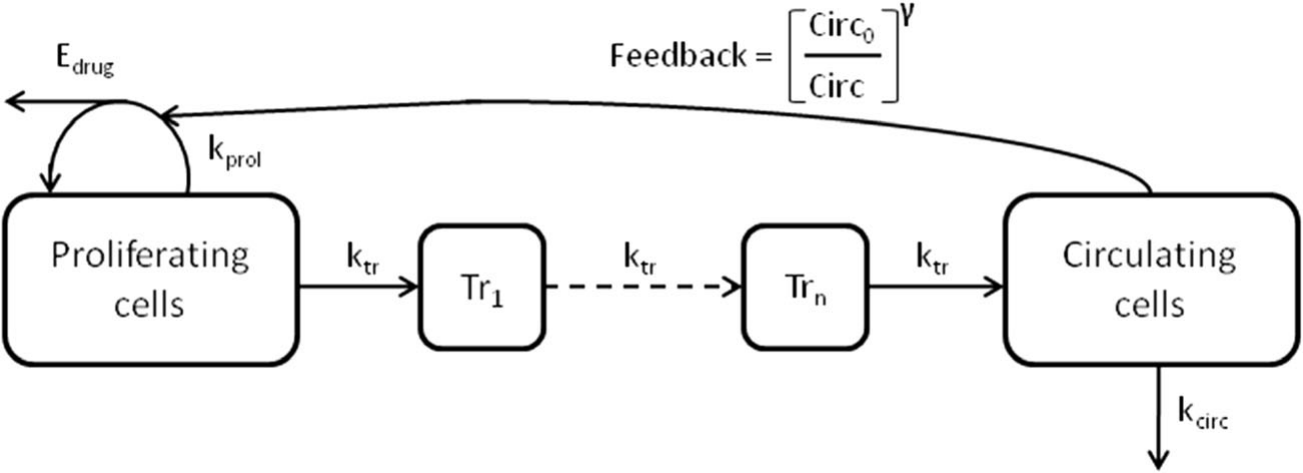
General model structure for myelosuppression. *E*_*drug*_ drug effect, *k*_*tr*_ maturation rate constant, *k*_*prol*_ proliferation rate constant, *k*_*circ*_ degredation rate constant, *Tr*_*n*_ transition compartment, *Circ*_*0*_ circulating cells at baseline, *Circ* amount of circulating cells, and *γ* factor for impact of feedback

Currently, a more mechanistic modeling approach is used. Mechanistic models mimick the physiological processes of hematopoiesis. Generally, this improves the predictive value of the model, since the mechanism-related parameters represent actual physiological processes. Hematopoiesis is characterized by proliferation of progenitor cells in the bone marrow, followed by maturation and degradation of blood cells [13]. To make useful models for pharmacokinetic and pharmacodynamic (PK-PD) analysis, several simplified semi-mechanistic models have been developed (Table 1) [14–19]. These semi-mechanistic models are all characterized by a proliferation cell compartment or progenitor compartment containing cells that have self-renewing capacity and a compartment representing circulating cells. In order to account for the maturation process, that delays the effect of the drug, either lag time or one- to multiple-transit compartments are added to the model structure. In some of the semi-mechanistic models, a feedback loop is incorporated to describe the rebound of blood cells, exceeding the blood count at baseline, which occurs when drug concentrations decrease. Typically, this feedback effect is driven by the amount of circulating blood cells, which affects the rate of proliferation in the progenitor compartment. Drug effects were modeled to affect the proliferation rate or the amount of progenitor cells. Model characteristics of five published semi-mechanistic models are summarized in Table 1. A general model structure for myelosuppression is depicted in Fig. 1.

**Table 1:** Semi-mechanistic models describing blood count over time

Reference		Drug	Observed variable	Par^a^	Tr^b^	k_prol_^c^	Drug effect
Minami (1998)	[14]	Paclitaxel	WBC	4	Lag time	Zero order	*E*_max_
Friberg (2000)	[15]	DMDC	ANC	7	9	Zero order	*E*_max_
Zamboni (2001)	[16]	Topotecan	ANC	4	1	Zero order	*E*_max_
Friberg (2002)	[17]	Docetaxel, etoposide, and paclitaxel	ANC WBC	5	3	First order	Linear
Panetta (2003)	[18]	TMZ	ANC	5	2	First order	*E*_max_
Bulitta (2009)	[19]	Paclitaxel paclitaxel EL	ANC	5	1^d^	Zero order	Linear

*WBC* white blood cell count, *ANC* absolute neutrophil count, *TMZ* temozolomide

^a^Number of parameters estimated in pharmacodynamic model

^b^Number of transit compartments or if lag time is used

^c^Proliferation rate constant

^d^Maturating pool of cells

The first semi-mechanistic model developed used a two-compartment indirect response model to describe the time course of leucopenia in paclitaxel- and etoposide-treated patients [14]. The drug inhibited the proliferating cells only during a sensitive stage. This model is the only model that used lag time to mimick the maturation process instead of using transit compartments.

In 2000, Friberg et al. published a semi-mechanistic model, modeling the absolute neutrophil counts (ANCs) in 2′-deoxy-2′-methylidenecytidine (DMDC)-treated patients [15]. This model contained three additional proliferating compartments and five non-mitotic compartments. The first-order elimination from the first progenitor compartment was proportional to the DMDC concentration. A fraction of the effect of DMDC on the first progenitor compartment was added to the other proliferating compartments. The non-mitotic compartments were not affected by DMDC concentration. Cytotoxic anti-cancer drugs only affect proliferating cells; therefore, this is a more elegant way of incorporating the maturation chain and the delay in drug effect as compared to using lag time.

The most well-known semi-mechanistic model for myelosuppression by Friberg et al. published in 2002 is often referred to as the golden standard for modeling the time course of myelosuppression [17]. Development was based on data from docetaxel-, etoposide-, and paclitaxel-treated patients, in whom ANC and WBC were measured. The model structure is described by the following equations:

$$ \frac{dProl}{dt}={k}_{prol}\cdot {P}_{cells}\cdot \left(1-{E}_{drug}\right)\cdot {\left(\frac{Cir{c}_0}{Cir}\right)}^{\gamma }-{k}_{tr}\cdot {P}_{cells} $$

$$ \frac{dTransit1}{dt}={k}_{tr}\cdot {P}_{cells}-{k}_{tr}\cdot Transit1 $$

$$ \frac{dTransit2}{dt}={k}_{tr}\cdot Transit1-{k}_{tr}\cdot Transit2 $$

$$ \frac{dTransit3}{dt}={k}_{tr}\cdot Transit2-{k}_{tr}\cdot Transit3 $$

$$ \frac{dCirc}{dt}={k}_{tr}\cdot Transit3-{k}_{circ}\cdot Circ $$

where *k*_prol_ represents the first-order proliferation input, *P*_cells_ represents the amount of cells in the proliferation compartment, and *E*_drug_ represents the drug effect. The feedback mechanism was described by (Circ_0/_Circ)^*γ*^, where Circ_0_ is the ANC or WBC blood count at baseline, Circ is the amount of circulating blood cells, and *γ* is the parameter estimate to determine the impact of the feedback. Transits 1–3 represent the amount of cells in the transit compartments, and *k*_tr_ represents the rate constant between compartments. Degradation of circulating cells is described by the rate constant *k*_circ_. The first-order proliferation input is different from previously described models, which used a zero-order rate constant of proliferation. It was assumed that the proliferation, maturation, and degradation rate constants were equal. Therefore, only three system-related parameters were estimated. An analysis was conducted to evaluate the consistency of the system-related parameters, by fixing them and re-estimating the drug-related parameters. The drug-related parameter estimates were comparable. Additionally, the system-related parameter estimates were similar for different drugs, enabling interchangeability of the model between drugs. In 2003, a model with similar characteristics was published [18].

The most recent model for myelosuppression is a multiple-pool life span model for neutropenia [19]. This model estimates the life span of cells staying in a certain stage, starting with duration of the cells in the progenitor compartment, followed by duration in the maturation compartment, and lastly by duration in the circulation until degradation of the neutrophils. The model requires extensive computing with use of 17 differential equations.

In conclusion, the model published by Friberg et al. in 2002 is most frequently used and has several advantages above the other models [17]. This model has a clear separation between drug-related and system-related parameters, making the model applicable to different drugs. Additionally, the model only estimates few system-related parameters, allowing it to model sparse data sets. Four of the reported models in this review have been compared to the model by Friberg et al. using ANC data from patients treated with a Plk-1 inhibitor [20]. The results of this analysis implicated that none of the models showed superior performance to the model by Friberg et al. [17, 20]. Lastly, this model has been used in multiple studies with different drugs and research aims and has also been modified and applied to describe thrombocytopenia in patients treated with cytotoxic anti-cancer drugs or targeted therapies [21–33]. The extensive application, the limited number of system-related parameters, and the overall experience with this model make it the best starting point for modeling myelosuppression.

### Cardiovascular adverse effects

For both cytotoxic and targeted therapies, cardiovascular toxicity has been reported. Anthracyclines can cause arrhythmias during or after administration and chronic cardiac toxicity, resulting in irreversible left ventricular dysfunction and congestive heart failure (CHF) [34]. Anthracycline-induced CHF has been related to dose, where patients receiving a cumulative dose of 550 mg/m^2^ doxorubicin were at increased risk of developing CHF [35]. Trastuzumab has also been associated with cardiac complications, inducing (reversible) left ventricular systolic dysfunction, which can result in CHF [36]. Additionally, tyrosine kinase inhibitors, targeting the vascular-endothelial growth factor receptor (VEGFR), have been associated with hypertension and cardiac arrhythmias as well as other systemic anti-cancer drugs [37–39].

#### Hypertension

Pharmacodynamic models have been developed for lenvatinib- and sunitinib-induced hypertension, describing the change in blood pressure (BP) over time in relation to treatment (Table 2) [40, 41]. The relationship between lenvatinib exposure and increase of diastolic (d) and systolic (s) BP was best described by an indirect response model with two effect models for dBP and sBP. The plasma concentration of lenvatinib at the time point of BP measurement was used as input rate for the indirect effect model with a linear function [40]. Additionally, this model included the effect of anti-hypertensive therapy on blood pressure. A similar indirect response model is used to describe the increase of dBP in sunitinib-treated patients [41].

**Table 2:** Pharmacodynamic models describing continuous and categorical adverse effects

Reference		Drug		AE^a^	Observed variable	Par^b^	Drug effect
Continuous adverse effects							
van Hasselt (2011)		[42]	Trastuzumab	Cardiotoxicity	LVEF	3	*E*_max_
Keizer (2010)		[40]	Lenvatinib	Hypertension	BP	3	Linear
Hansson (2013)		[41]	Sunitinib	Hypertension	dBP and sBP	3	Linear
Marostica (2015)		[43]	Moxifloxacine	QT prolongation	QTc	4	Linear
Categorical adverse effects							
Keizer (2010)		[40]	Lenvatinib	Proteinuria	CTC	6	Linear
Hénin (2008)		[48]	Capecitabine	HFS	CTC	10	*E*_max_
Hansson (2013)		[41]	Sunitinib	HFS and fatigue	CTC	12	*E*_max_
Suleiman (2015)		[49]	Erlotinib	Rash and diarrhea	CTC	6	Linear

*HFS* hand-foot syndrome, *CTC* NCI-CTC-AE, *LVEF* left ventricular ejection fraction, *BP* blood pressure, *d* diastolic, *s* systolic, *QTc* heart rate-corrected QT interval

^a^Adverse effect

^b^Number of model parameters estimated in structural pharmacodynamic model (fixed effects excluding drug effect parameters)

#### Cardiotoxicity

Cardiotoxicity as expressed as decline in left ventricular ejection fraction (LVEF) was used to develop a pharmacodynamic model with an effect compartment model to describe the decrease in LVEF over time, related to trastuzumab exposure [42]. Recovery of the LVEF was implemented in the model. Additionally, the model incorporated the prior cumulative anthracycline dose as a covariate and found that this dose was an important determinant for the sensitivity to LVEF decline.

The relation between exposure and increase in BP and decrease of LVEF, as reported in both papers, are empirical models [40, 42]. It is, therefore, difficult to extrapolate these models directly between different drugs that might induce hypertension.

#### QT interval prolongation

Anti-cancer drugs, such as anthracyclines and tyrosine kinase inhibitors, can prolong the QT interval, which can lead to severe cardiac arrhythmias, such as torsade de pointes [38]. Concentration-QT modeling can provide important information on the relation between exposure and heart rate-corrected QT interval (QTc) [43, 44]. However, these models are mainly developed for anti-arrhythmic drugs and not for anti-cancer drugs. A recent publication investigated the effect of moxifloxacine, a compound that prolongs the QT interval, by developing a PK-PD model for translational purposes [43]. The time course of the QT interval is described by the following three components: the individual heart rate correction, the circadian rhythm, and the drug effect:

$$ QT={\left(Q{T}_0\cdot \frac{RR}{R{R}_{ref}}\right)}^{\alpha }+A\cdot \cos \left(\frac{2\pi }{24}\left(t-\varnothing \right)\right)+{E}_{drug} $$

where QT_0_ represents the QT interval at baseline, RR is the heart rate, and RR/RR_ref_ is multiplied by QT_0_ to correct for individual heart rate. *A* and ø represent the amplitude and the phase of the circadian rhythm, respectively, and ED represent the drug effect. Subsequently, the probability of QT prolongation above a critical threshold (e.g., >10 or >20 ms) can be derived. An effect compartment can be considered for modeling the ECG time course [45]. Similar model structures could be used to model QT prolongation induced by anti-cancer drugs.

### Ordered categorical adverse effects

Typically, cancer therapy-related adverse effects are graded using the ordered NCI-CTC-AE scale, ranging from 0 to 5. This range represents no adverse effects (0) to mild, moderate, severe, life-threatening adverse effects, and lastly death (5). Adverse effects such as vomiting, diarrhea, rash, fatigue, and hand-foot syndrome (HFS) are solely described by this ordered categorical scale. A conventional approach to describe the relation between exposure and the occurrence of a certain grade is by statistically comparing the incidence of grades between different dose groups. Early models for these type of adverse effects used ordered logistic regression or proportional odds models [46, 47]. Both comparing the incidence of adverse effects and the ordered logistic regression approach have shortcomings. In these analyses, only the most severe grade of the adverse effect observed in a patient is used. By comparing incidences, the already categorized data is dichotomized, leading to substantial loss of information and ignoring the time course of the effect. Furthermore, the dependency of the previous observed grade in predicting the probability of the occurrence of the next grade is not taken into account. This problem can be addressed by implementing a Markov process in the model. First-order Markov models take into account the value of the preceding observation. The proportional odds model can be extended with a first-order Markov model [48]. In this way, the probability of transition between severity grades of adverse effects depends on the preceding grade. Typically, the logit transformation is used to constrain values of probabilities between 0 and 1, similar to the logistic regression approach. This approach has been used for modeling different anti-cancer-induced graded adverse effects (Table 2).

HFS has been described by a proportional odds model with a Markov process to model the cumulative probabilities of getting a grade 0, 1, or ≥2 for HFS related to accumulation of capecitabine [49]. HFS and fatigue in sunitinib-treated patients have been modeled using a first-order Markov model that was similar to the extension of the proportional odds model [41]. Vascular endothelial growth factor receptor 3 (VEGFR-3) was identified as biomarker, and its relative change over time was modeled as predictor of the occurrence and severity of fatigue and HFS.

Keizer et al. used a Markov transition model to describe proteinuria in patients treated with the VEGFR inhibitor lenvatinib [40]. Using a compartmental structure, each adverse event grade is represented with a compartment, which in turn is denoted with its own differential equation. The probability of experiencing a certain grade is represented by the corresponding compartment amount. The amounts in all compartments sum up to 1 at any time. At each observation, these amounts are re-set to a full probability for the observed state and 0 for all other states, and hence, a first-order Markov property is introduced. The rate constants for the movement of these amounts (i.e., probabilities) between compartments, which reflect the transitions between the different grades, are then estimated. As Markov models potentially allow transition between all states in the model, assumptions can be made to reduce the number of parameters to be estimated. For this reason, in the analysis of Keizer et al., only the transitions between neighboring grades were estimated. The following differential equations were used:

$$ \frac{dP\left(G{r}_0\right)}{dt}={k}_{10}\cdot P(1)-{k}_{01}\cdot P(0) $$

$$ \frac{dP\left(G{r}_1\right)}{dt}={k}_{01}\cdot P(0)+{k}_{21}\cdot P(2)-{k}_{10}\cdot P(1)-{k}_{12}\cdot P(1) $$

$$ \frac{dP\left(G{r}_2\right)}{dt}={k}_{12}\cdot P(1)+{k}_{32}\cdot P(3)-{k}_{21}\cdot P(2)-{k}_{23}\cdot P(2) $$

$$ \frac{dP\left(G{r}_3\right)}{dt}={k}_{23}\cdot P(2)-{k}_{32}\cdot P(3) $$

**Fig 2:**
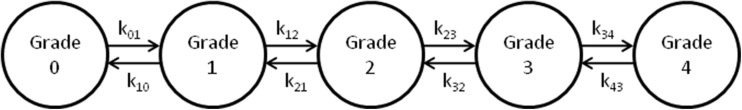
General structure of Markov model. *Amount in compartment* probability and *k*_*xx*_ rate constants between probabilities [40, 50]

Recently, a modeling and simulation framework for erlotinib-induced rash and diarrhea in patients with NSCLC was published [50]. The model structure was similar to the model used by Keizer et al., which was a continuous-time Markov model. A general structure of a Markov model, incorporating 5 grades (0-4), is depicted in Fig. 2.

The use of Markov processes is preferred over use of the proportional odds model for modeling ordered graded adverse effects. Markov models allow use of total longitudinal data on graded toxicities over time. Furthermore, these models take the preceding grade observed into account, which enables the precise characterization of the dynamics of toxicity. The Markov models currently published are empirical models. More mechanistic elements can easily be introduced in these models, for instance, using latent variables describing the underlying pharmacodynamic effects. However, this underlying mechanism is in most cases unknown. The major drawback of analyzing graded or categorical data is the fact that information is lost by using categories. In some cases, there is not a sufficient number of observations of severely graded adverse effects. Therefore, grades are sometimes merged together, leading to loss of already categorized information. However, if the clinical relevance between merged grades is not profound, this is an acceptable approach. Though, if available, the underlying observations might be better than the use of grades (e.g., blood pressure, instead of grades for hypertension).

## Application of adverse effect models

Developed models for adverse effects have been applied to support decision-making regarding treatment optimization and clinical development. Ideally, PK-PD modeling frameworks are developed that integrate data on pharmacokinetics, adverse effects, and efficacy. An example of such a framework is available for sunitinib [41]. This paper did not only include modeling of ANC, fatigue, blood pressure, and HFS but also investigated if adverse effects were predictive for overall survival. Hypertension and neutropenia were found predictive for overall survival, functioning as biomarkers for treatment response.

Adverse effect models can additionally support decision-making regarding dose adjustments and dose individualizations, using simulation methods. The previously described modeling and simulation framework for erlotinib-induced rash and diarrhea investigated the safety of high-dose erlotinib pulses (1600 mg/week + 50 mg/day remaining week days) proposed, compared to the standard dose (150 mg/day) and different other dosing regimens [50]. Based on a simulation analysis using the framework developed, severe rash was predicted to occur in 20 % of patients treated with the pulsed dosing regimen, compared to 12 % in patients treated with the standard dosing regimen. In contrast with the common perception, radiotherapy was found to attenuate erlotinib-induced rash significantly, which advocates for using erlotinib and radiotherapy together. The framework also included a survival model, finding that experiencing rash at any grade was associated with improved clinical efficacy in terms of survival, albeit not significantly. Another example demonstrated that modeling can be helpful for determining individual dose adjustment of capecitabine to reduce severe grade HFS while maintaining efficacy [51]. The paper reports a clinical trial simulation in which the proportional odds Markov model was used on individual patient data [49]. Intolerable HFS (grade ≥2) was predicted for the next treatment cycle, based on the previous cycle for each patient. Dose adjustments were made accordingly. Individualized dose adjustments using the Markov model were compared to using standard dose adjustments and found to reduce the duration of intolerable HFS by 10 days without loss of efficacy. Both modeling frameworks are examples of how a modeling approach can support dose adjustments and dose individualizations using predictive simulation methods.

Lastly, modeling and simulation of adverse effect models can optimize treatment and support clinical trial designs. The hypertension model, discussed in this review, has been used to optimize treatment with lenvatinib [52]. This paper investigated four strategies to clinically manage lenvatinib-induced hypertension to maximize both the number of patients on treatment and the average dose level during treatment, with use of simulations. An adverse effect-guided dose titration could potentially increase drug exposure without additional toxicity. Additionally, a design where anti-hypertensive treatment was followed by lenvatinib dose reduction proved to keep a large number of patients on treatment. This approach aimed at minimizing treatment cessation due to toxicity in order to improve response to treatment. The intervention designs were supportive of development of a phase II clinical trial.

## Discussion

This review reports several structural models for modeling adverse effects of anti-cancer drugs. Firstly, the best modeling approach depends on the type of data available and secondly, on whether or not prior knowledge of the underlying mechanism of the adverse effect is available. If the adverse effect is reported as continuous variable and prior knowledge on the mechanism behind the effect, the best approach is to develop a mechanism- or semi-mechanism-based model. Mechanistic models generally have a better predictive performance and potentially allow for extrapolation beyond the conditions on which the model was developed. The model by Friberg et al., published in 2002, proves to be the best starting point for modeling hematologic toxicity, with only few pharmacodynamic parameters to be estimated, and the mechanistic approach makes the model interchangeable between different anti-cancer drugs [17]. If no prior mechanistic knowledge is available or implementation in the model is impossible, data can be modeled using generic pharmacodynamic effect models. This more empirical approach can give insight in how an adverse effect evolves over time, if data is continuous. In some cases, the underlying continuous measurement is graded using the NCI-CTC-AE scale. For example, hypertension can be graded as such, however, the underlying continuous measurement, blood pressure, is needed to grade this toxicity. Therefore, blood pressure measurement itself can be used to develop PK-PD models. In conclusion, if an underlying continuous measurement is available, this longitudinal continuous data is preferred over ordered graded data, since it is less prone to loss of information. In subsequent simulation studies, the clinically well-accepted graded score can still be derived from the continuous data. Adverse effects like diarrhea, vomiting, and HFS are difficult to quantify and are described by ordered categorical grades. In this case, the best approach is to model the probabilities using a proportional odds model with a Markov process. The probability of a certain grade will then depend on the previously observed grade, which is true for almost all observed effects in oncology.

Modeling and simulation methods for analyzing adverse effects are preferred over the conventional comparison of adverse effect incidences between dosing groups. Quantitative models consider the variability between patients, allowing integration of patient characteristics that might be important in predicting the safety profile. Patient characteristics can alter systemic exposure to the drug and may lead to differences in onset, severity, and duration of adverse effects. Typically, physiological factors such as age, body size, gender, kidney function, and liver function can alter exposure, as well as pharmacogenetic factors and administration of other drugs [53]. Integration of these patient characteristics can be helpful in managing individual dose adaptations. In addition, proposed models can be used to model adverse effects driven by combination therapy, which is often applied in the oncology setting.

Established PK-PD models can predict different clinical scenarios. These simulations are particularly helpful in finding the optimal relationship between exposure and safety. Ideally, a PK-PD modeling framework is developed, that integrates data on exposure, efficacy, and toxicity, to assess the optimal balance between safety and efficacy [41, 54]. Modeling tumor growth as a biomarker for efficacy can be of added value in assessing this balance [55].

In conclusion, mathematical modeling of adverse effects can provide insight in how toxicities evolve over time and if or what patient-related factors can impact this time course. In addition, a modeling approach includes all available data, minimizing loss of information as is typically the case using more conventional methods of analyzing toxicity data. At last, modeling and simulation frameworks have been proven to support clinical trial designs, to optimize treatment, and to guide dose adjustments or dose individualizations. Therefore, modeling adverse effects proves to be a helpful tool for both improvement of clinical management and support of decisions regarding drug development.
